# Evaluation of analytical models to estimate depth of fluorescence objects in biological media

**DOI:** 10.1117/1.JBO.31.2.026003

**Published:** 2026-02-23

**Authors:** Héctor A. García, Hang M. Nguyen, Caleb Y. Kwon, Samuel S. Streeter, Veronica C. Torres, Mayna H. Nguyen, Ethan P. M. LaRochelle, Alberto J. Ruiz, Eric Henderson, Scott C. Davis, Kimberley S. Samkoe

**Affiliations:** a Thayer School of Engineering, Dartmouth College, Hanover, New Hampshire, United States; b CIFICEN (UNCPBA - CICPBA - CONICET), Buenos Aires, Argentina; c Geisel School of Medicine, Dartmouth College, Hanover, New Hampshire, United States; d Oregon Health and Science University, Portland, Oregon, United States; e QUEL Imaging, LLC, Hartford, Vermont, United States; f Dartmouth-Health, Lebanon, New Hampshire, United States

**Keywords:** fluorescence, turbid media, biological tissue, inclusions, depth imaging, near-infrared spectroscopy, optical properties, Monte Carlo simulations, phantoms, indocyanine green

## Abstract

**Significance:**

Performing a ratiometric analysis of the fluorescence signals noninvasively measured at two different wavelengths can provide depth estimates of subsurface inner structures in a simple and fast manner, allowing for real-time applications in clinical settings. This can be done using the initially proposed single-excitation–multiple-emission wavelengths approach or by implementing a modified multiple-excitation–single-emission approach; the latter being sometimes preferred due to the larger variation of tissue optical properties at shorter wavelengths. However, previous works validating this method with Monte Carlo (MC) simulations, experiments on tissue-mimicking phantoms, and *in vivo* measurements on small animal models have reported different degrees of accuracy.

**Aim:**

We tested the influence of factors not generally accounted for in the analytical model used for data interpretation (e.g., tissue geometry and boundaries, inclusion size and shape, and spectral characteristics of the excitation source). To address these limitations, we developed an improved theoretical framework that explicitly accounts for these factors during data interpretation.

**Approach:**

Model validation was carried out with MC simulations and with phantom experiments using indocyanine green as the fluorescence contrast agent. The aimed tissue optical properties were those characteristic of the prostate in a wide range of wavelengths (from 550 to 900 nm).

**Results:**

The aforementioned factors have a strong influence when changing the original single-excitation–multiple-emission approach to a multiple-excitation–single-emission approach. Though this might make the latter a less preferable method, the low variability of the optical properties in the multiple emission approach (as it happens with prostate tissue) negatively impacts the depth reconstruction process.

**Conclusions:**

Regardless of the ratiometric strategy employed, accurate depth estimation requires that the theoretical model closely replicate the experimental conditions. Careful matching of model assumptions to the measurement environment is essential to achieve reliable data interpretation.

## 1 Introduction

Iatrogenic injuries to critical, normal anatomical structures (nerves, ureters, vessels, ducts, glands) during surgery are devastating complications, leading to substantial morbidity—sometimes mortality—in patients. Injuries to these structures often arise due to their close proximity to the surgical target. Furthermore, local pathological anatomy necessitating surgery may complicate normal structure identification, especially when structures are buried at depth. Peripheral nerve injury is estimated to occur in 0.16% to 10.8% of all patients undergoing robotic-assisted procedures,^1^ with substantially higher incidences (upward of 30%) of subjects reporting urinary and fecal incontinence and sexual dysfunction after lower abdominopelvic surgeries—including radical prostatectomies,^2^^,^^3^ colorectal surgeries,^4^^,^^5^ and gynecological surgeries.^6^

Prostate cancer is the most common solid organ malignancy diagnosed in men.^7^ In fact, one in eight men will be diagnosed with prostate cancer in their lifetime, and one in 36 will die from the disease. Despite most prostate cancers being detected at an early stage and thus curable, the morbidity associated with these treatments can be severe. Specifically, surgical removal of the prostate—radical prostatectomy, the most common treatment for clinically localized prostate cancer—is associated with a high risk of erectile dysfunction,^8^ which can be life-changing and debilitating for men undergoing cancer treatment. The cause of erectile dysfunction is injury to branches of the cavernosal nerves that traverse the outer edge of the prostate and are easily damaged during surgery. Part of the reason for this injury is the challenge in identifying these nerves and their accompanying blood vessels, highlighting the need to identify both nerve and vascular tissue during robot-assisted, laparoscopic radical prostatectomy.

Over the last few decades, the use of optical imaging methods has gained increasing interest in biology and medicine due to their noninvasiveness, simplicity, ability for continuous monitoring, and inexpensive costs when compared with other established technologies.^9^^–^^14^ In particular, the use of light sources and detectors in the near infrared (NIR) window—between ∼650 and ∼900  nm—allows for the detection of inhomogeneities surrounded by bulk tissue, as well as deep photon penetration, thanks to the relatively low light absorption of biological components such as hemoglobin, melanin, fat, and water.^15^^–^^17^ Based on this, many optical techniques, including NIR spectroscopy, have been developed to accurately sense chromophore and fluorophore distributions in tissues and organs;^18^^–^^20^ however, high scattering in the NIR strongly reduces the spatial resolution, hindering these technologies from accurately assessing the size, depth, and margins of tumors and critical normal structures, both of which must be handled with great care but paradoxically must be removed and preserved, respectively.

The use of fluorescence contrast agents can overcome some of the natural limitations of NIR spectroscopic methods by enhancing the contrast of targeted regions inside tissue,^21^ especially by exploiting a fluorophore’s spectral characteristics, which can provide valuable information about its spatial distribution and kinetics. As fluorescence-guided surgery has become more popular, a number of NIR fluorescence dyes have been used and/or developed for identifying normal critical structures that are concealed in the surgical field. Indocyanine green (ICG) has been extensively used during angiography to identify blood vessels and blood flow in surgical tissues.^22^ In addition, a number of NIR dyes have been developed for the identification of other normal structures, such as ureters,^23^^–^^25^ glandular tissue,^26^^,^^27^ and nerves.^28^^,^^29^

In terms of data interpretation and modeling, in 2005, Swartling et al.^30^ developed a technique to accurately quantify the depth of a fluorescent layer embedded in an optically turbid medium based on the ratiometric analysis of the fluorescence signal measured at two different wavelengths. Later on, other works explored the same approach with different types of inclusions, fluorophores, and experimental configurations.^31^^–^^34^ In 2011, Leblond et al.^35^ developed a simple yet elegant theoretical model based on the diffusion approximation (DA) to the radiative transfer equation (RTE)^36^^,^^37^ that links the object’s depth with the ratio of the fluorescence signals at two emission wavelengths. This approach is attractive because of its simplicity and speed, enabling real-time applications in clinical settings. However, subsequent works validating this method with Monte Carlo (MC) simulations, experiments on tissue-mimicking phantoms, and *in vivo* measurements on small animal models have reported different degrees of accuracy.^38^^–^^42^ The reason for this is the following: although the original theoretical model assumes a point-like fluorescent inclusion illuminated by a point-like excitation source, both of which are embedded in an infinite medium without external boundaries, and measuring at two different emission wavelengths; none of the subsequent studies followed these assumptions (especially the one related to the infinite medium, which is impossible to achieve in actual clinical settings). Therefore, although wide-field fluorescence imaging allows the identification of such structures buried within the surgical field, analytical depth information is not always obtainable to guide the surgeon while making critical incisions.

In this work, we developed a new theoretical model to determine the depth of a fluorescent object embedded in tissue and compared its performance with the model introduced by Leblond et al.^35^ Specifically, we used MC simulations and phantom experiments to evaluate depth recovery while varying tissue geometry and boundaries, inclusion size and shape, and spectral characteristics of the excitation source. In addition, we examined these models under the original single-excitation–multiple-emission approach (as reported in Refs. 30, 31, and 35) and compared it with the case of a multiple-excitation–single-emission approach, as implemented, for example, in Refs. 41 and 42. Our results suggest that, for fluorescent objects embedded in media with tissue-like optical properties, the model that best matches the experimental and simulation conditions achieves depth reconstruction errors of ≤1  mm for depths up to 10 mm. In summary, ratiometric analysis of fluorescence signals is able to provide accurate depth estimates of fluorescent objects in optically turbid media, as long as the appropriate model is implemented for data interpretation.

In the following, only combinations of two wavelengths (one target and one reference) will be evaluated; besides, we will refer to the single-excitation–multiple-emission approach simply as “dual-emission,” and the multiple-excitation–single-emission approach will be referred to as “dual-excitation.”

## 2 Materials and Methods

### 2.1 Theoretical Modeling

#### 2.1.1 Dual-emission approach

**Fig. 1:**
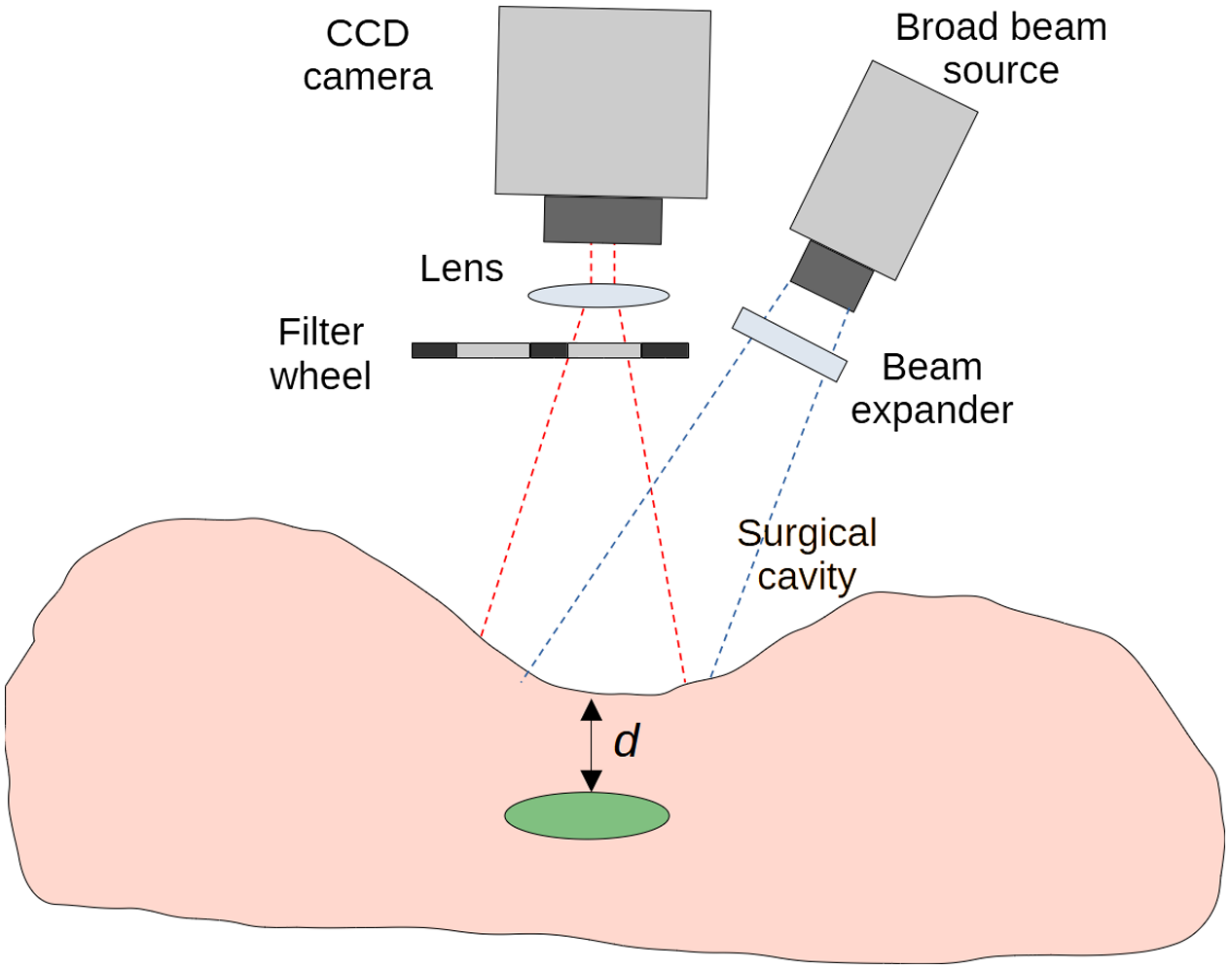
Schematic representation of an experimental setup for fluorescence ratio measurements in the context of a fluorescence-guided surgery.

As first demonstrated by Leblond et al.,^35^ the depth d of an object embedded in biological tissue (Fig. 1) can be retrieved by measuring the fluorescence signal at two different emission wavelengths $\lambda_{1}^{m}$ and $\lambda_{2}^{m}$, that is, $I_{1}^{m}(d)=I(d,\lambda_{1}^{m})$ and $I_{2}^{m}(d)=I(d,\lambda_{2}^{m})$, taking the ratio and computing the natural logarithm:

$$\ln[\frac{I_{1}^{m}(d)}{I_{2}^{m}(d)}]\equiv \ln[\Gamma^{m}(d)]=(\frac{1}{\delta_{2}^{m}}-\frac{1}{\delta_{1}^{m}})\times d+\ln(\frac{D_{2}^{m}}{D_{1}^{m}}),$$ (1)

where $\delta =\sqrt{D/\mu_{ab}}$ is the effective penetration depth, $D=[3(\mu_{ab}+\mu_{sb}^{\prime }){]}^{-1}$ is the diffusion coefficient, and $\mu_{ab}$ and $\mu_{sb}^{\prime }$ are the background absorption and reduced scattering coefficients, respectively; the subscripts 1 and 2 correspond to the wavelengths $\lambda_{1}$ and $\lambda_{2}$, respectively, and the superscript m explicitly states that these are emission wavelengths. Equation (1) provided the first theoretical explanation for the results reported by Swartling et al.^30^ and Svensson and Andersson-Engels.^31^ Other studies used this model for depth reconstruction purposes based on fluorescence ratiometric measurements, not only under the dual-emission approach but also under the dual-excitation approach.^38^^,^^40^^–^^44^

**Fig. 2:**
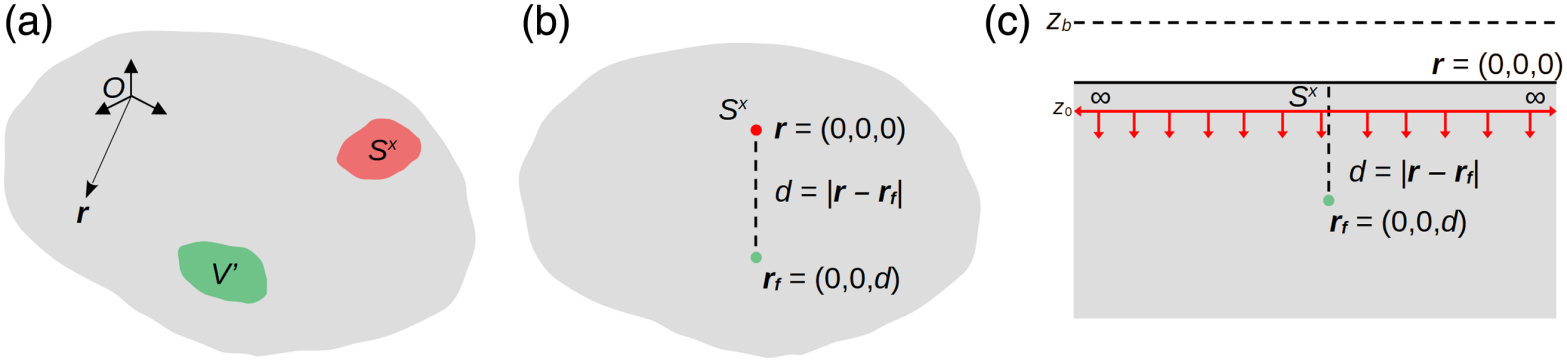
Schematic representations of different configurations of an optically turbid medium with a light source and fluorescent inclusion. (a) Representation utilizing an excitation source $S^{x}$ and a volume of fluorophore $V^{\prime }$ embedded. Excitation and emission fields [$\psi^{x}(r)$ and $\psi^{m}(r)$, respectively] are measured at position r relative to the coordinate system *O*. (b) Point-like excitation source $S^{x}$ illuminating a point-like fluorescent inclusion at a distance $d=|r-r_{f}|$ in an infinite turbid medium; **r** = (0, 0, 0) refers both to the position of the excitation source and the observation point, whereas $r_{f}\;=(0,0,d)$ refers to the position of the fluorescent inclusion. (c) Planar excitation source illuminating the fluorescent inclusion from the top surface of a semi-infinite medium, with the fluence vanishing at the extrapolated boundary $z_{b}=2AD$; though the physical position of the source is r=(0,0,0), the diffusion approximation shifts its vertical coordinate to $z=z_{0}=1/\mu_{s}^{\prime }$.

Now, we will focus on how Eq. (1) can be derived because this highlights the assumptions involved in the approaches studied here. Let’s assume an excitation source $S^{x}(r)=S(r,\lambda^{x})$ (at the excitation wavelength $\lambda^{x}$) illuminating an arbitrary volume of fluorophore $V^{\prime }$ inside the turbid medium [Fig. 2(a)]. If the photon migration process takes place under a diffuse regime, the fluorescence emission and excitation fields $\psi^{m}(r)=\psi (r,\lambda^{m})$ and $\psi^{x}(r)=\psi (r,\lambda^{x})$, respectively, are related to each other by a system of coupled diffusion equations:^43^^,^^45^

$$-D^{x}\nabla^{2}\psi^{x}(r)+\mu_{a}^{x}\psi^{x}(r)=S^{x}(r),$$ (2)

$$-D^{m}\nabla^{2}\psi^{m}(r)+\mu_{a}^{m}\psi^{m}(r)=Q_{f}\mu_{af}^{x}\psi^{x}(r),$$ (3)

where $Q_{f}$ is the fluorophore’s quantum yield, $\mu_{af}$ is the fluorophore’s absorption coefficient, the superscript x refers to excitation, and the superscript m refers to emission; in addition, the variable **r** refers to the observation point. Here, it must be noted that $\mu_{a}=\mu_{ab}+\mu_{af}$, and $\mu_{af}=\varepsilon_{f}C_{f}$, being $\varepsilon_{f}$ the fluorophore’s molar extinction coefficient (or, in other words, the excitation spectrum) and $C_{f}$ its concentration. The right-hand side of the emission Eq. (3) acts as a source of fluorescent light, which allows expression of $\psi^{m}(r)$ as:^35^

$$\psi^{m}(r)=Q_{f}\varepsilon_{f}^{x}(\lambda^{m})\int_{V^{\prime }}d^{3}r^{\prime }\psi^{x}(r^{\prime })C_{f}(r^{\prime })G^{m}(r,r^{\prime }),$$ (4)

where $G^{m}(r,r^{\prime })$ is the Green’s function of the diffusion equation at $\lambda^{m}$ for a point-like source. Here, we explicitly indicate that $C_{f}$ is a function of position to allow for arbitrary fluorophore distributions inside the turbid medium; for instance, if the source of fluorescence is totally concentrated at position $r_{f}$ [$C_{f}(r)=C_{f}\delta (r-r_{f})$], Eq. (4) reduces to:

$$\psi^{m}(r,r_{f})=Q_{f}\varepsilon_{f}^{x}(\lambda^{m})C_{f}\psi^{x}(r_{f})G^{m}(r,r_{f}).$$ (5)

Next, with the help of Eq. (5), we can compute the ratio of $\psi^{m}(r,r_{f})$ at two different emission wavelengths, leading to:

$$\Gamma^{m}(r,r_{f})=\frac{\psi_{1}^{m}(r,r_{f})/\varepsilon_{f}^{x}(\lambda_{1}^{m})}{\psi_{2}^{m}(r,r_{f})/\varepsilon_{f}^{x}(\lambda_{2}^{m})}=\frac{G_{1}^{m}(r,r_{f})}{G_{1}^{m}(r,r_{f})}.$$ (6)

Of note, according to this last expression, the fluorophore’s excitation spectrum evaluated at each emission wavelength, $\varepsilon_{f}^{x}(\lambda^{m})$, can be thought of as a calibrating factor (although other methods exist as well for calibrating the fluorescence fields^30^^,^^31^^,^^35^^,^^38^^,^^42^).

At this point, we can use the Green’s function for a point-like fluorescence source in an infinite medium:^46^

$$G_{\infty }^{m}(d)=\frac{e^{-d/\delta^{m}}}{4\pi D^{m}d},$$ (7)

where $d=|r-r_{f}|$ [Fig. 2(b)]; inserting Eq. (7) into Eq. (6) and taking the natural logarithm automatically lead to Eq. (1). The assumptions followed to this point are: (i) the DA is valid (i.e., photons undergo several scattering events before being either absorbed or detected, $\mu_{sb}^{\prime }\gg \mu_{ab}$); (ii) the fluorophore distribution can be represented as an infinitely small point, and background fluorescence is completely neglected (permitting the approximation from Eqs. (4) to (5); (iii) the turbid medium is infinite [allowing use of Eq. (7) to reach Eq. (1)]. Changing any of these conditions will change the underlying math, consequently modifying the form of Eq. (1). For example, in the same paper by Leblond et al., the case where the infinite medium is replaced by a semi-infinite medium [Fig. 2(c)] is discussed, yielding this result:^35^

$$\Gamma_{1/2}^{m}(d)=\Gamma_{\infty }^{m}(d)\times [1-\frac{\exp(-4{AD}_{1}^{m}/\delta_{1}^{m})}{1+4{AD}_{1}^{m}/d}]\times [1-\frac{\exp(-4{AD}_{2}^{m}/\delta_{2}^{m})}{1+4{AD}_{2}^{m}/d}{]}^{-1},$$ (8)

with A being a function of the refractive indices of the medium and the environment.^37^ Here, it can be clearly seen that the natural logarithm of Eq. (8) does not follow a simple linear relationship with d, except for two limiting cases:

$$\Gamma_{1/2}^{m}\to \Gamma_{\infty }^{m},\;d\to 0\;\Gamma_{1/2}^{m}\to \Gamma_{\infty }^{m}\times \frac{1-e^{-4{AD}_{1}^{m}/\delta_{1}^{m}}}{1-e^{-4{AD}_{2}^{m}/\delta_{2}^{m}}},\;d\gg 4A\;\max\{D_{1}^{m},D_{2}^{m}\};$$ (9)

for typical tissue optical properties, the second case holds when d≫2 mm.^15^^,^^17^^,^^35^ Moreover, the derivation of Eq. (8) requires an extra assumption: the detector must be placed exactly on top of the inclusion, which requires accurate prior knowledge of its lateral position. In any case, the simplicity of Eq. (1) has always been preferred over the complexity of the ratio given by expression Eq. (8).^38^^–^^42^

#### 2.1.2 Dual-excitation approach

In this section, we will derive the new model. We can start by noting an interesting feature of Eq. (6): this expression is independent of the type of excitation source (geometry, wavelength, directionality) used to activate the fluorophore. This is a direct consequence of working under the dual-emission approach, for which $\psi^{x}(r_{f})$ appears both in the numerator and the denominator of the ratio with the same $\lambda^{x}$, hence canceling out. When switching to the dual-excitation approach, Eq. (6) is modified:

$$\Gamma^{m}(r,r_{f})=\frac{\psi_{1}^{x}(r,r_{f})}{\psi_{2}^{x}(r,r_{f})},$$ (10)

such that now the characteristics of the excitation source need to be taken into account. It can also be noted that $G^{m}(r,r_{f})$ disappears because only one $\lambda^{m}$ is used, but this does not mean that the information about the fluorophore’s distribution is completely lost; on the contrary, Eq. (10) is still derived from Eq. (5), which was already obtained by assuming a point-like fluorophore distribution.

In the special case, in which the excitation source is point-like [Fig. 2(b)]:

$$S^{x}(r)=S_{0}(\lambda^{x})\delta (r)$$ (11)

[where $S_{0}(\lambda^{x})$ is the wavelength-dependent output power], the excitation fluence for an infinite medium takes the form:

$$\psi_{\infty }^{x}(r)=\int_{V^{\prime }}d^{3}r^{\prime }S^{x}(r^{\prime })G_{\infty }^{x}(r,r^{\prime })=S_{0}(\lambda^{x})G_{\infty }^{x}(r),$$ (12)

where now $G_{\infty }^{x}(r,r^{\prime })$ is the Green’s function for a point-like excitation source, which happens to take the same form as Eq. (7). With this in mind, taking the natural logarithm of Eq. (10) yields an equation of the same form as Eq. (1) [after properly normalizing by $S_{0}(\lambda^{x})$]. Let us summarize this in the following way: although the model introduced by Leblond et al. is independent of the excitation source under the dual-emission approach, in the dual-excitation approach, it only holds when the excitation source is point-like.

Another very common type of excitation source is the epi-illumination configuration.^47^^–^^49^ This can be combined with the more realistic semi-infinite medium, in which case, the mathematical form of the source needs to be changed from Eq. (11) to:

$$S^{x}(r)=S_{0}(\lambda^{x})[\delta (z-z_{0}^{x})-\delta (z+z_{0}^{x}+2z_{b}^{x})],$$ (13)

with $z_{0}^{x}=1/\mu_{sb}^{\prime x}$, whereas $z_{b}^{x}=2AD^{x}$ is the extrapolated boundary at which the fluence vanishes [Fig. 2(c)], assuming the extrapolated boundary condition (EBC) holds.^37^^,^^50^ Next, we can use Eq. (13), together with the Green’s function for a semi-infinite medium:^35^

$$G_{1/2}^{x}(r,r^{\prime })=\frac{1}{4\pi D^{x}}[\frac{e^{-|r-r_{s}^{\prime }|/\delta^{x}}}{\left|r-r_{s}^{\prime }\right|}-\frac{e^{-|r-r_{i}^{\prime }|/\delta^{x}}}{\left|r-r_{i}^{\prime }\right|}],$$ (14)

(where $\left|r-r_{s}^{\prime }|=|d-z_{0}^{x}\right|$ and $|r-r_{i}^{\prime }|=d+z_{0}^{x}+2z_{b}^{x}$) to compute $\psi^{x}(r)\equiv \psi^{x}(d)$ according to the integral in Eq. (12):

$$\psi_{1/2}^{x}(d)=\frac{\delta^{x}}{D^{x}}e^{-(d+z_{b})/\delta^{x}}\sinh(\frac{z_{b}^{x}-z_{0}^{x}}{\delta^{x}})\;d\leq z_{0}^{x}\;\psi_{1/2}^{x}(d)=\frac{\delta^{x}}{D^{x}}e^{-(d+z_{b})/\delta^{x}}\sinh(\frac{z_{b}^{x}+z_{0}^{x}}{\delta^{x}})\;d>z_{0}^{x}.$$ (15)

These two cases arise due to the presence of the absolute value $\left|d-z_{0}^{x}\right|$ in Eq. (14); this requires prior knowledge of the object’s depth, which in general, is not readily available. Hence, we can take the second of the two conditions in Eq. (15) (inevitably at the cost of losing some accuracy at shallow depths, typically up to 0.5 mm) to calculate the natural log ratio at two different excitation wavelengths:

$$\ln[\Gamma_{1/2}^{m}(d)]=(\frac{1}{\delta_{2}^{x}}-\frac{1}{\delta_{1}^{x}})\times d+\ln[\frac{\delta_{1}^{x}D_{2}^{x}\sinh(\frac{z_{01}^{x}+z_{b1}^{x}}{\delta_{1}^{x}})}{\delta_{2}^{x}D_{1}^{x}\sinh(\frac{z_{02}^{x}+z_{b2}^{x}}{\delta_{2}^{x}})}].$$ (16)

This new model, similar to the case of the original model given by Eq. (1), still presents a linear relationship between the log ratio and the target’s depth; however, the main difference is the form of the y− intercept. In addition, we note that under the DA $\mu_{sb}^{\prime }\gg \mu_{ab}$, which means that D can be approximated reasonably without considering the absorption coefficient, i.e., $D=(3\mu_{sb}^{\prime }{)}^{-1}$^37^^,^^51^^–^^53^; this redefinition impacts the y− intercept as well as the slope of Eq. (16).

### 2.2 Monte Carlo Simulations

To validate the new theoretical model developed under the dual-excitation approach, as well as to analyze the similarities and differences between the dual-excitation and the dual-emission approaches, MC simulations were run using MCXLAB, a Matlab-based toolbox for MC simulations in optically turbid media.^54^^,^^55^ The simulations ran on a workstation with an Intel Core i9 14th Gen. CPU, an NVIDIA RTX 4000 GPU and 128 GB RAM. Diffuse fluorescence images at the top surface of the modeled medium were simulated for different excitation (600, 640, 680, 720, and 760 nm) and emission (822, 837, 851, 867, and 889 nm) wavelengths; these images served as the raw data needed to feed the models previously introduced to estimate the depth of a fluorescent inclusion. More specifically, the generation of the synthetic diffuse fluorescence data consisted of a two-simulation process: first, an MC simulation was run at one excitation wavelength, and then, the information of the absorbed photons was used to run a second simulation in which the source of light (at the emission wavelength) was the fluorophore distribution inside the medium.^56^^,^^57^

**Fig. 3:**
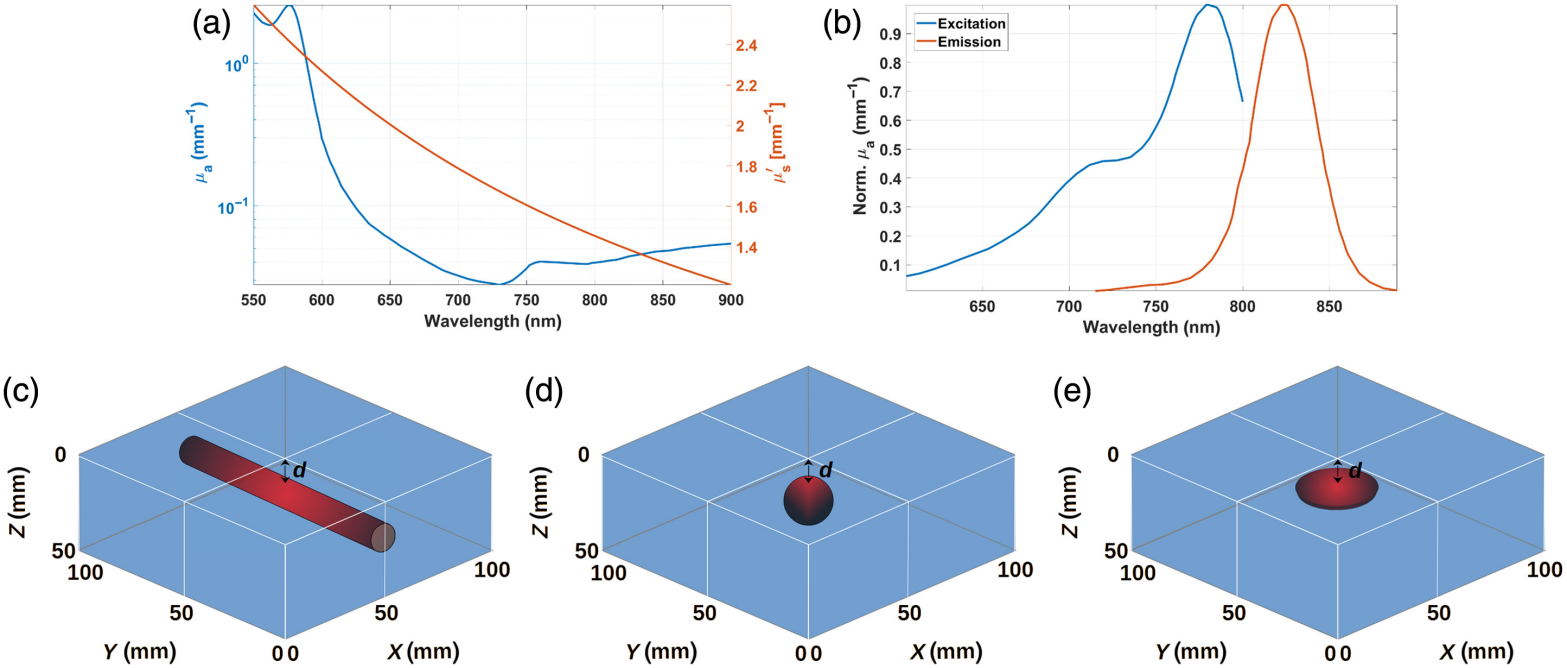
Optical properties and MC simulation configurations. Top row: absorption and reduced scattering coefficients spectra for prostate tissue used in the MC simulations (a) and ICG’s excitation and emission spectra (b) used in the MC simulations. Bottom row: schemes (not to scale) depicting the cylindrical (c), spherical (d), and disk (constant thickness of 1 mm) (e) inclusions embedded at a depth d from the top surface (z=0  mm) of the turbid medium; in all these cases, the medium was illuminated from the top (either with a point-like or a planar source).

Unless otherwise stated, each simulation consisted of launching $10^{8}$ photons into a medium with dimensions of $100\times 100\times 50\;{\mathrm{mm}}^{3}$ with a voxel size of 0.5 mm. One of the main applications of the technique studied here is maximizing normal tissue sparing in lower abdominopelvic surgeries,^2^^,^^3^ so we limited the range of optical properties to typical values for tissues and organs found in that region of the body; in particular, we simulated the prostate’s absorption and reduced scattering behaviors^17^ [Fig. 3(a)] for the background medium. ICG in a concentration of 1  μM was used to fill the inclusion’s volume, yielding optical properties $\mu_{a,\mathrm{inclusion}}(\lambda )=\mu_{a,\mathrm{prostate}}(\lambda )+\mu_{a,\mathrm{ICG}}(\lambda )$ and $\mu_{s,\mathrm{inclusion}}^{\prime }(\lambda )=\mu_{s,\mathrm{prostate}}^{\prime }(\lambda )$; ICG was chosen because it is a clinically approved fluorescent agent^58^ and commonly used in angiography to identify cylindrical vascular structures. The excitation and emission spectra of ICG in dimethyl sulfoxide (DMSO) were measured at QUEL Imaging LLC, providing a maximum excitation peak at 779 nm and a maximum emission peak at 820 nm^59^ [Fig. 3(b)]. These spectra were used in accordance with Eq. (6) to calibrate the MC diffuse fluorescence images.

As previously stated, one of the major assumptions in the development of the ratiometric models herein is that the fluorescent object is infinitely small. However, in real clinical scenarios, the targeted anatomical structures might be different sizes (from a few tenths or hundreds of microns to a few millimeters) and geometries (spherical, cylindrical, disks, or more arbitrary in the general case). Hence, we performed a series of MC simulations using different types of inclusions [Figs. 3(c)–3(e)], with radii ranging from r=0.5  mm to 5 mm, and at depths d=0 to 10 mm; in these studies, we defined d as the shortest distance between the top surface of the medium and the inclusion’s boundary.

### 2.3 Phantom Imaging Experiments

#### 2.3.1 Phantom preparation

Phantom molds were fabricated by 3D printing with optically absorbing black polylactic acid (PLA) plastic (40 mm wide × 50 mm long × 30 mm deep). Cylindrical inclusions were chosen over sphere or disk inclusions to approximate the appropriate target anatomy (e.g., nerves and blood vessels). Each mold contained 1-mm diameter through-holes such that a capillary tube could be inserted at a constant depth. Unique molds were created such that the inclusions were at different nominal depths (0.5, 1.0, 1.5, 3.0, 5.0, and 7.0 mm).

**Fig. 4:**
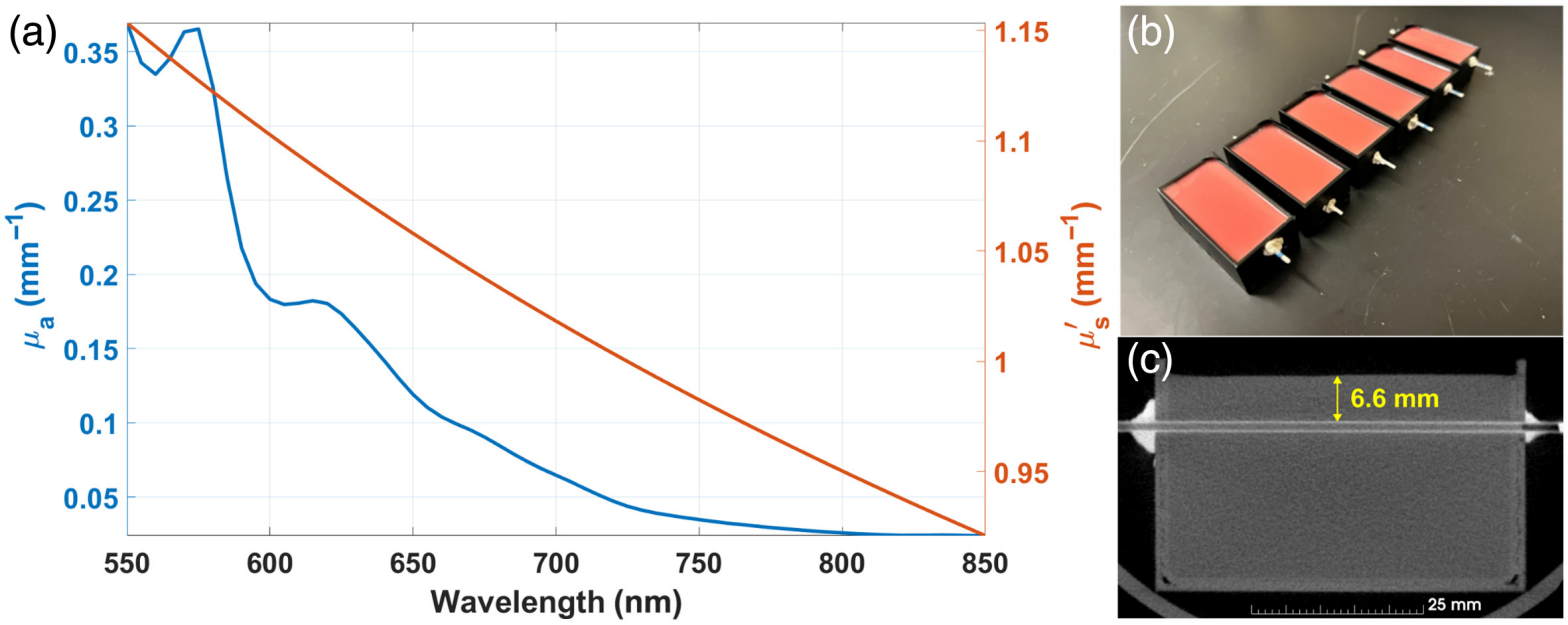
Experimental phantom composition. (a) Phantoms’ background absorption and reduced scattering coefficients. (b) Set of phantom molds with capillary tubes at different depths. (c) Capillary tube depth confirmation using micro-computed tomography.

The capillary tubes were filled with 1  μM ICG (250 mg, US Pharmacopeia, Rockville, Maryland, United States) together with 3% *v*/*v* intralipid and 5% *v*/*v* bovine serum albumin. The capillary tubes were placed in the molds, and the holes were sealed with glue. To simulate the optical properties of the background prostate tissue^17^ [Fig. 4(a)], gelatin phantoms were prepared with a recipe consisting of 2% *v*/*v* blood (bovine whole blood, Lampire Biological Laboratories, Lampire, Pipersville, Pennsylvania, United States), 3% *v*/*v* intralipid (Baxter Healthcare Corporation, Deerfield, Illinois, United States), 10% *w*/*v* gelatin from porcine skin (G2500, Sigma-Aldrich, St. Louis, Missouri, United States), and 10% yeast extract (09182, Sigma-Aldrich).^60^ Yeast was introduced to tune the oxy-to-deoxyhemoglobin ratio through oxygen consumption. The gelatin mixture (37°C) was transferred into the 3D-printed molds [Fig. 4(b)] and placed in a 4°C refrigerator to solidify. This procedure helped reproduce prostate-like optical properties as given in Ref. 17; specifically, the reduced scattering coefficient spectrum was obtained with the parameters given in Table 1 from that reference, whereas the absorption coefficient spectrum was computed with the parameters listed in Table 3 from the same reference.

Once solidified, the phantoms immediately underwent fluorescence imaging followed by confirmation of fluorescent inclusion (i.e., capillary tube) depth by micro-computed tomography scanning (80  μm resolution, Quantum GX3, Revvity, Waltham, Massachusetts, United States); Fig. 4(c) shows an example of depth confirmation for a capillary tube of 7 mm nominal depth. From the same gelatin mixture, a set of 3-, 4-, and 5-mm thick, square samples was created; these samples were used to measure the optical properties of the gelatin mixture (i.e., $\mu_{ab}$, $\mu_{sb}^{\prime }$).

#### 2.3.2 Optical property measurements

Optical property measurements were performed using a commercial dual-beam precision benchtop spectrophotometer (Lambda 1050+ with 150 mm integrating sphere, PerkinElmer, Waltham, Massachusetts, United States). The spectrophotometer measured transmittance and reflectance of each constant-thickness gelatin sample (3, 4, and 5 mm). Transmittance and reflectance data for each sample were in turn fed into an inverse adding-doubling (IAD) algorithm to generate point-based optical property spectra (i.e., $\mu_{ab}$, $\mu_{sb}^{\prime }$) for the prostate-mimicking gelatin mixture.^61^^,^^62^

IAD-based optical property estimates were completed in triplicate (one set of spectra for each constant-thickness sample) and then averaged to generate the final optical properties used in all experimental dual-excitation results in this study, producing the curves shown in Fig. 4(a). IAD algorithm assumptions included an anisotropy (*g*) of 0.8 and an index of reflection (n) of 1.3.

#### 2.3.3 Fluorescence imaging

Fluorescence images were collected using a custom setup. The illumination source was a supercontinuum laser (SuperK Blue, NKT Photonics, Denmark) with a tunable line filter (SuperK Varia, NKT Photonics, Denmark) to select the wavelength band. Light was delivered from the tunable line filter through an optical fiber to a fly’s eye array, which spread the light over the imaging field of view (∼100  mm×∼100  mm). Images were captured using a cooled scientific complementary metal-oxide-semiconductor (sCMOS) camera (Edge 4.2 bi, Excelitas PCO GmbH, Kelheim, Germany) with a two-lens setup for an effective focal length of 50 mm and a bandpass filter to isolate ICG fluorescence (832 nm center wavelength, 37 nm bandwidth, 18-401, Edmund Optics Barrington, New Jersey, United States). The supercontinuum laser, tunable line filter, and sCMOS camera were controlled through a custom LabView interface (Labview 2018, National Instruments, Austin, Texas, United States).

Fluorescence images of each phantom were acquired at the following excitation wavelengths with a full-width half-maximum bandwidth of ∼10  nm: $\lambda_{x}=[640,740,750,760]\;\mathrm{nm}$. Camera settings were constant for all acquisitions, and the position of each phantom was fixed during imaging. In addition, fluorescence images of a 2 mL sample of exposed ICG solution (identical to that in each capillary tube) were acquired; these images provided the necessary calibration measurements for the dual-excitation depth retrieval [(as shown in Eq. (6)].

#### 2.3.4 Fluorescence depth prediction

All fluorescence images were first background-subtracted. Each phantom fluorescence image was then normalized by the median pixel value from the calibration well signal at the same excitation wavelength. After calibration, maps of the natural log of fluorescence ratios at two excitation wavelengths [$\ln(I_{1}/I_{2})$] were generated. Effective optical properties at each excitation wavelength were based on average property values within a 10-nm-wide bandwidth centered on each target excitation wavelength. Each $\ln(I_{1}/I_{2})$ map and the corresponding effective optical properties ($\mu_{ab1}$, $\mu_{ab2}$, $\mu_{sb1}^{\prime }$,$\mu_{sb2}^{\prime }$) were fed into the analytical models [i.e., Eq. (1) for the original model, or Eq. (16) for the updated model]. The output from this process was a single dual-excitation-based depth estimation map. From each depth map, a 10-mm line profile centered over the embedded capillary tube was extracted, and descriptive pixel statistics were recorded (i.e., pixel mean and standard deviation).

## 3 Results

### 3.1 Validation with MC Simulations

#### 3.1.1 Influence of the excitation source’s characteristics

**Fig. 5:**
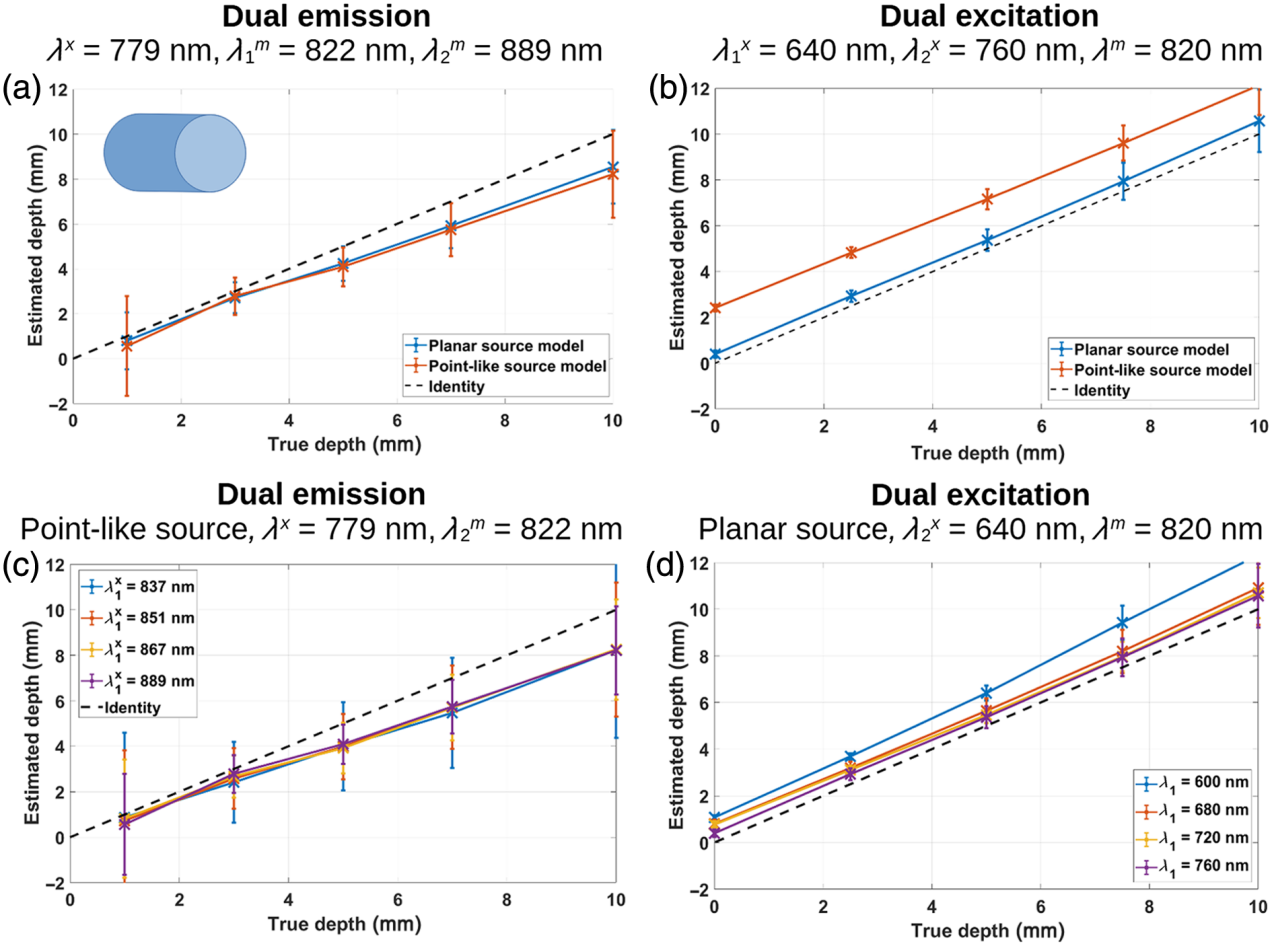
Comparison of depth reconstruction from MC simulated data using the dual-emission and dual-excitation models. (a) Depth reconstruction under the dual-emission approach and epi-illumination, using the original point-like source model (orange line) and the planar source model (blue line); $\lambda^{x}=779\;\mathrm{nm}$, $\lambda_{1}^{m}=822\;\mathrm{nm}$, $\lambda_{2}^{m}=889\;\mathrm{nm}$. (b) Depth reconstruction under the dual-excitation approach and epi-illumination, using the original point-like source model (orange line) and the planar source model (blue line); $\lambda_{1}^{x}=640\;\mathrm{nm}$, $\lambda_{2}^{x}=760\;\mathrm{nm}$, $\lambda^{m}=820\;\mathrm{nm}$. (c) Depth reconstruction under the dual-emission approach and point-like illumination, using the original point-like source model with $\lambda^{x}=779\;\mathrm{nm}$ and $\lambda_{1}^{m}=822\;\mathrm{nm}$, for different target wavelengths. (d) Depth reconstruction under the dual-excitation approach and epi-illumination, using the updated model with $\lambda_{2}^{x}=640\;\mathrm{nm}$ and $\lambda^{m}=820\;\mathrm{nm}$, for different target wavelengths. In all cases, the inclusion is a cylinder of 1 mm radius [inset shown in subplot a)].

Figure 5(a) shows the estimated depth versus the true depth for a cylindrical object (1 mm radius) when planar excitation is used under the dual-emission approach. The orange curve corresponds to the reconstruction with the theoretical model developed for epi-illumination [Eq. (16)], whereas the blue curve represents the reconstruction using the original model that assumes a point-like excitation [Eq. (1)]. Here, it can be easily noticed that the difference in performances is negligible. On the contrary, under the dual-excitation approach [Eq. (16)], the geometrical characteristics of the source have a strong impact on the depth reconstruction [Fig. 5(b)].

Next, Fig. 5(c) depicts the depth retrieval for the cylinder in the dual-emission approach for the point-like source, by choosing target (emission) wavelengths of 837, 851, 867, and 889 nm, and fixing $\lambda_{1}^{m}=822\;\mathrm{nm}$. The results are almost independent of $\lambda_{1}^{m}$ likely due to the small differences in the prostate’s optical properties in that region of the NIR spectrum [see Fig. 3(a)]; this could also explain the depth underestimation as the object is placed deeper into the medium. In the dual-excitation approach performed with planar illumination [Fig. 5(d)], fixing $\lambda_{2}^{x}=640\;\mathrm{nm}$ and combining with target excitation wavelengths of 600, 680, 720, and 760 nm yield more distinguishable depth estimates, especially for $\lambda_{1}^{x}=600\;\mathrm{nm}$. This last combination overestimates the object’s depth by 1 to 2 mm (in the whole range of actual depths), whereas the best performance is achieved with $\lambda_{1}^{x}=760\;\mathrm{nm}$. This behavior can be explained by considering how similar or different the optical properties of the prostate are at 600, 640, and 760 nm. At 600 nm $\mu_{a}$ is rather high, hindering the penetration of photons into deep tissue (hence worsening the results as the object is deeper) and also deviating the photon migration process from the diffuse regime.

From these last two subplots, it is possible to see that in most tissues, where absorption is dominated by oxy- and deoxyhemoglobin,^15^^,^^17^ the dual-excitation approach is preferred because it provides a better sensitivity than the dual-emission approach in a very narrow bandwidth.

#### 3.1.2 Effect of inclusion size and geometry

**Fig. 6:**
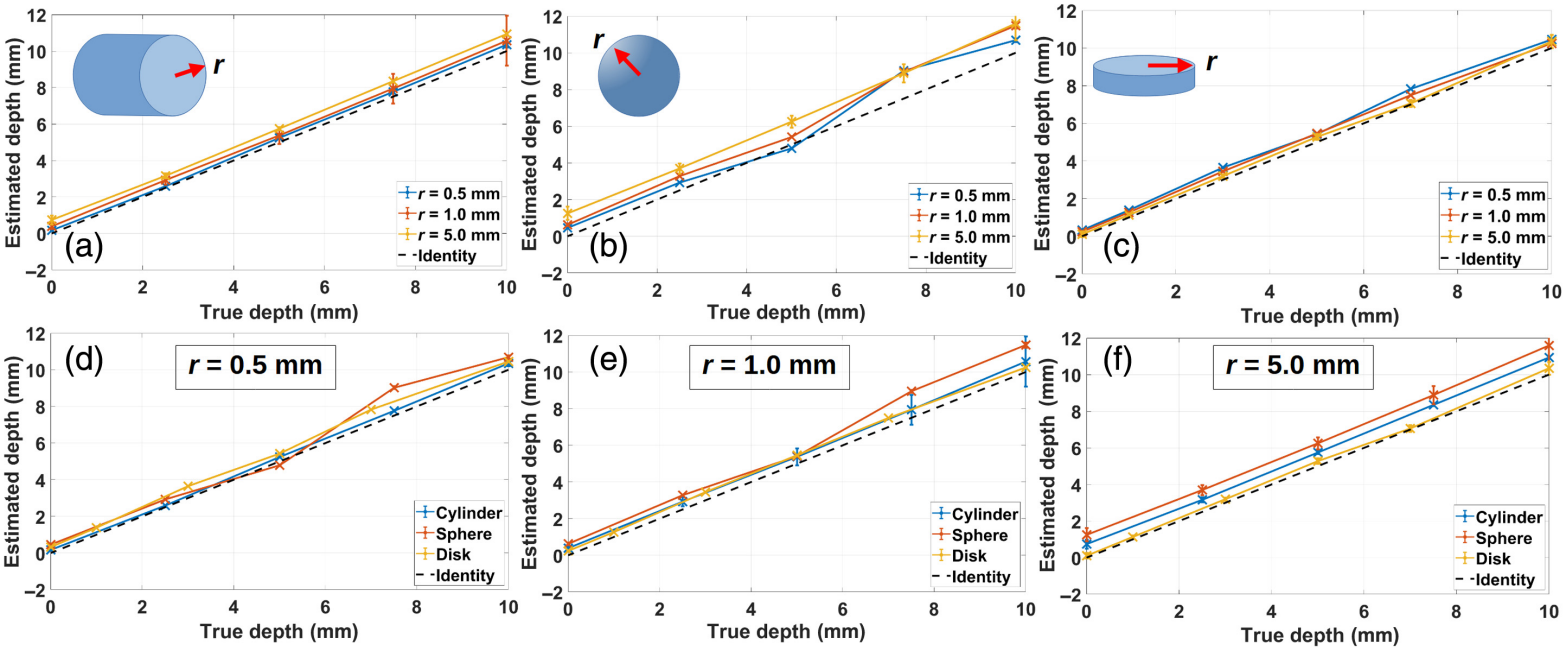
Top row: depth reconstruction under the dual-excitation approach with planar illumination for (a) a cylinder, (b) a sphere, and (c) a disk of varying radii (inclusion’s geometry included as an inset in the corresponding subfigure for better guidance). Bottom row: depth reconstruction under the dual-excitation approach with planar illumination for a cylinder, a sphere and a disk of radius (d) 0.5 mm, (e) 1 mm, and (f) 5 mm. In all cases, $\lambda_{1}^{x}=640\;\mathrm{nm}$, $\lambda_{2}^{x}=760\;\mathrm{nm}$. All cases correspond to MC simulated data.

Though the theoretical models assume a point-like fluorescent inclusion, the depth estimates are obtained within reasonable error for finite (i.e., nonpoint-like) objects. In Figs. 6(a) (cylinder) and 6(b) (sphere), the retrieval worsens as the radius increases; on the contrary, in Fig. 6(c) (disk), the retrieval improves with increasing radius.

Figures 6(d)–6(f) show the same results as before, but rearranged to compare the effect of the inclusion’s geometry on the depth estimates. As the radius of the object is increased from 0.5 mm [Fig. 6(d)] to 5 mm [Fig. 6(f)], the curves corresponding to the cylinder, the sphere, and the disk are separate from each other. This means that the dependence of the model on the object’s geometry relies on its size; in other words, the smaller the object, the less effect its geometry has on the reconstruction.

#### 3.1.3 Effect of input optical properties

Depth estimations by means of fluorescence ratiometric techniques assume accurate knowledge of the background optical properties. However, this could be a potential challenge in the context of real-time data acquisition, especially during surgery, due to variable factors (patient-specific tissue morphology and composition, difficult access of optical devices to the region of interest, bleeding, etc.). Hence, some degree of inaccuracy is expected for the measured optical properties, which in turn will have an impact on the depth reconstruction. The following analysis is focused solely on the dual-excitation approach for a cylindrical inclusion.

**Fig. 7:**
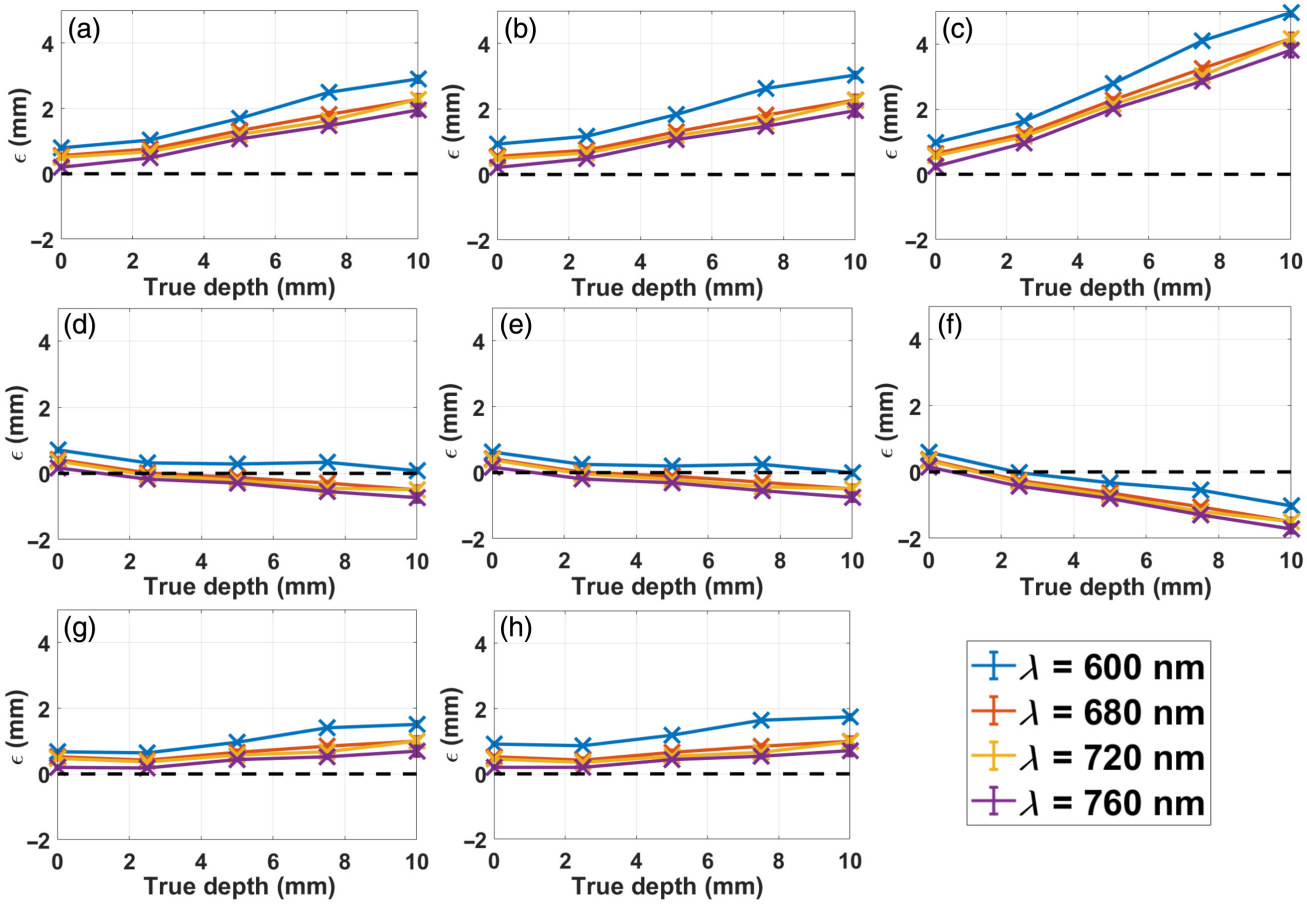
Depth estimate error ϵ versus true depth for the case of a cylinder (r=0.5  mm) under the dual-excitation approach using epi-illumination, with $\lambda_{2}^{x}=640\;\mathrm{nm}$. (a) 25% underestimation in $\mu_{a}$; (b) 25% underestimation in $\mu_{s}^{\prime }$; (c) 25% underestimation in both optical properties; (d) 25% overestimation in $\mu_{a}$; (e) 25% overestimation in $\mu_{s}^{\prime }$; (f) 25% overestimation in both optical properties; (g) 25% underestimation in $\mu_{a}$, 25% overestimation in $\mu_{s}^{\prime }$; (h) 25% overestimation in $\mu_{a}$, 25% underestimation in $\mu_{s}^{\prime }$. All cases correspond to MC simulated data.

We started by studying the effect of underestimating the optical properties by 25%. Figure 7(a) shows the error $\epsilon =d_{\mathrm{est}}-d_{\mathrm{true}}$, when only the absorption coefficient is inaccurate; Fig. 7(b) shows the same quantity when the inaccuracy is produced just on $\mu_{s}^{\prime }$; and Fig. 7(c) shows the case in which both optical properties are simultaneously inaccurate. As can be seen, the overall effect of underestimated optical properties is an overestimation of the object’s depth. The largest impact is produced for simultaneous inaccuracies in both coefficients (almost 50% error at 10 mm depth), followed by the cases with individual inaccuracies just in $\mu_{s}^{\prime }$ or just in $\mu_{a}$ (∼25% error at 10 mm depth).

Next, we analyze the situation in which the optical properties are overestimated by 25% [Figs. 7(e)–7(g)]. In this case, the retrieved depths are underestimated [except for the shallowest depth, being the reason given in the paragraph following Eq. (15)]. As before, the worst scenario is the one in which both $\mu_{a}$ and $\mu_{s}^{\prime }$ are overestimated simultaneously (producing errors of up to 20% at 10 mm depth), whereas the individual overestimation of each parameter has a much lesser impact on the depth reconstruction (less than 15% at 10 mm depth).

Another feasible situation is one in which the errors in the optical properties have opposite signs [Figs. 7(g) and 7(h)]. Either with −25% error in $\mu_{a}$ and +25% error in $\mu_{s}^{\prime }$ or vice versa, the overall effect is an overestimation of inclusion’s depth, though with a smaller magnitude (less than 20%) than the ones obtained underestimating $\mu_{a}$ alone, $\mu_{s}^{\prime }$ alone or both at the same time.

### 3.2 Validation with Experiments on Phantoms

**Fig. 8:**
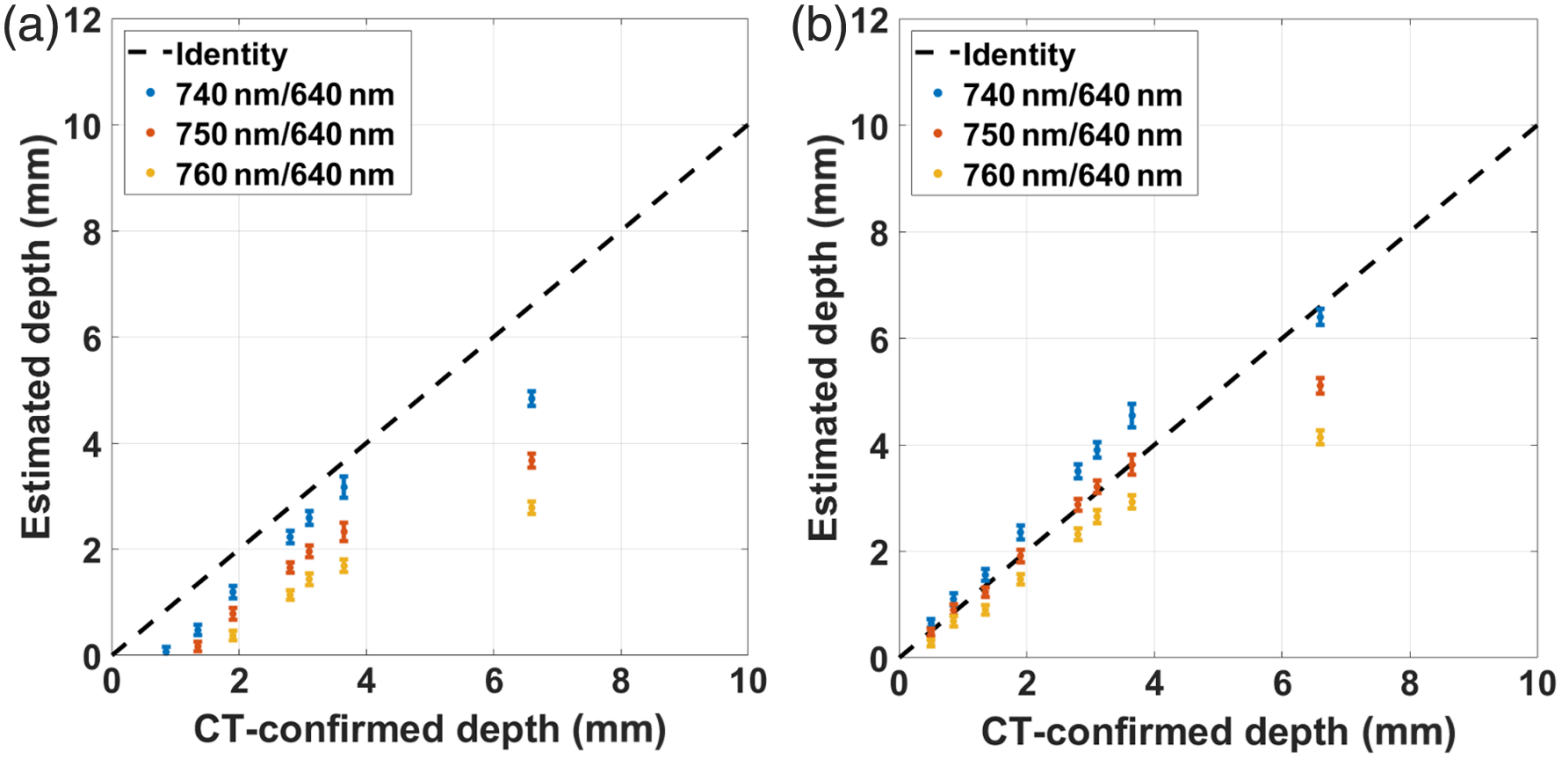
Dual-excitation-based depth estimation results from phantom experiments using (a) the original model given in Eq. (1) and (b) the updated model given in Eq. (16) introduced in this study. Depth estimates are shown as mean pixel values from a 10-mm profile within each constant-depth phantom ± one standard deviation.

The results for the experimental depth estimation under the dual-excitation approach, as detailed in Sec. 2.3, are shown in Fig. 8 for three different pairs of excitation wavelengths. The reconstruction using the original model [Eq. (1)] is depicted in Fig. 8(a), whereas the reconstruction performed with the updated model [Eq. (16)] is plotted in Fig. 8(b). In the first case, none of the tested wavelength pairs can follow the identity line, while in the second case, all the pairs accurately reproduce the identity line at shallow inclusion’s depths (up to 2 mm); for larger depths, the reconstructions distribute around the identity line with the pair 750 to 640 nm in the center, up to depths of ∼7  mm, when all the curves bend towards the CT-confirmed depth axis.

Overall, the updated model better matches the experimental conditions under which the capillary tube’s depth was measured, and hence, it should not be surprising that it better reproduces the actual depths.

## 4 Discussion

In this work, we expanded the theoretical framework introduced by Leblond et al.^35^ for depth reconstruction of fluorescent objects in biological tissues, being the motivation behind this fact that the underlying assumptions characterizing the original model (point-like source and inclusion, infinite tissue geometry lacking boundaries, dual-emission rather than dual-excitation measurements, etc.) are not always practical and/or achievable in real clinical scenarios. Specifically, we extended the theoretical formulation from a dual-emission approach to a dual-excitation approach, and we also developed a theoretical model for the case of a semi-infinite medium illuminated by a planar excitation source. Moreover, we demonstrated that, when switching from the dual-emission to the dual-excitation approach, factors such as the characteristics of the excitation source become relevant, meaning that they need to be accounted for to avoid losing accuracy in the depth estimation process.

The validation of the method was carried out with MC simulations using background media that mimicked the prostate’s optical behavior, given that one of the major goals of this study is to reduce the rate of iatrogenic injuries in the context of laparoscopic radical prostatectomies. The case of the prostate becomes relevant in this study particularly because its absorption coefficient presents a strong variation in the range from 550 to 800 nm (very suitable for the dual-excitation approach), whereas the variation in the range 820 to 890 nm is much lower, negatively impacting the depth reconstruction under the original dual-emission approach.

In addition, we studied the limitations of the theoretical models with parameters such as the geometry and the size of the inclusion. It must be noted that the models discussed in this paper assume an infinitely small source of fluorescence inside the medium, and therefore, departures from this condition are expected to introduce nuisances in the depth reconstruction. In the case of spherical and cylindrical inclusions, the depth retrieval worsened with increasing radii; for a disk inclusion, on the contrary, increasing its radius yielded better performance. Overall, it can be claimed that the size and the geometry can be combined into one single factor to produce a “vertical extension to lateral extension ratio”; when this parameter is small, the depth reconstruction is reasonably accurate. Nevertheless, this claim still needs to be formally proved by further refining the theoretical modeling.

Next, we analyzed the impact of feeding the theoretical model with wrong optical properties (under- and overestimations of 25%) on the depth retrieval. The worst scenario resulted in one in which both $\mu_{a}$ and $\mu_{s}^{\prime }$ are underestimated, producing depth deviations of up to almost 50% (for objects 10 mm deep) with respect to the true depth, whereas more bounded inaccuracies (±∼15%) are obtained when the optical parameters are overestimated or their errors have opposite signs. Nevertheless, available commercial systems for optical property measurements report uncertainties well below 25%,^63^^–^^65^ so we expect the method to perform much better than this in real case situations.

Additional validations were done with measurements on tissue-mimicking phantoms to test the impact of real experimental conditions on the depth reconstruction. When comparing the results between the depth retrieval with the original model and the updated model, the latter produced better estimates because it assumes illumination conditions much more similar to those characterizing the experimental device and the phantoms (wide-field illumination, multiple excitation wavelengths, semi-infinite medium). Even with this model, the depth recovery was very accurate for shallow depths (up to 3 mm) but deviated from the known depth values when the inclusion was deeper (with errors ranging from 5% to ∼40% depending on the wavelength pair). The cause of this is the limited sensitivity of the acquisition system, which, for depths large enough, barely notices differences between the fluorescence fields imaged at the reference and target wavelengths; here, the maximum achievable depth depends on different factors, such as the imaging system’s capabilities and the medium’s background optical properties.

Although sufficiently exhaustive in certain aspects, this study is subject to some important limitations. One of these is ignoring tissue background fluorescence (particularly when using ICG), which will likely reduce the contrast between the inclusion and the surrounding medium, consequently decreasing the performance of the method. Nevertheless, in cases where the fluorophore kinetics are very different between the inclusion and the background, this could potentially be overcome by measuring at a time at which this difference is maximized. It must also be noted that the models investigated here do not depend on the fluorophore concentration; however, in practical terms, the concentration governs the contrast between the target and the host medium, so it would be reasonable to expect a deterioration in the reconstruction process for small fluorophore concentrations.

## 5 Conclusion

In conclusion, accurate depth recovery of fluorescent objects embedded in tissue depends on theoretical models that faithfully reflect experimental conditions. The refinements introduced to the dual-excitation framework, including the new theoretical model, enhance its suitability for clinical translation, such as in radical prostatectomy, where wide-field illumination is used. Future efforts will extend validation to additional tissue types, evaluate contact versus noncontact illumination, and expand from dual-wavelength to multispectral (i.e., more than two wavelengths) implementations to further reduce uncertainties and improve depth estimations.

## Data Availability

The data that support the findings of this article are not publicly available but can be obtained upon reasonable request by contacting the corresponding author.
